# Is Exposure to Animal Feces Harmful to Child Nutrition and Health Outcomes? A Multicountry Observational Analysis

**DOI:** 10.4269/ajtmh.16-0270

**Published:** 2017-04-05

**Authors:** Derek Headey, Phuong Nguyen, Sunny Kim, Rahul Rawat, Marie Ruel, Purnima Menon

**Affiliations:** 1 Poverty, Health and Nutrition Division, The International Food Policy Research Institute (IFPRI), Washington, District of Columbia

## Abstract

It has recently been hypothesized that exposure to livestock constitutes a significant risk factor for diarrhea and environmental enteric disorder in young children, which may significantly contribute to undernutrition. To date, though, very little research has documented the extent of exposure to animal feces and whether this exposure is associated with child anthropometry in large samples and diverse settings. This study investigates these issues using data from the Alive and Thrive study conducted in rural areas of Ethiopia, Bangladesh, and Vietnam. These surveys used spot-checks to collect data on proxies of hygiene behaviors such as the cleanliness of mothers, young children, and the homestead environment, including the presence of animal feces. Animal feces were visible in 38–42% of household compounds across the three countries and were positively associated with household livestock ownership and negatively associated with maternal and child cleanliness. One-sided tests from multivariate least squares models for children 6–24 months of age indicate that the presence of animal feces is significantly and negatively associated with child height-for-age *z* scores in Ethiopia (β = −0.22), Bangladesh (β = −0.13), and in a pooled sample (β = −0.11), but not in Vietnam. There is also suggestive evidence that animal feces may be positively associated with diarrhea symptoms in Bangladesh. The results in this article, therefore, contribute to a growing body of evidence suggesting that animal ownership may pose a significant risk to child nutrition and health outcomes in developing countries.

## Introduction

As of 2011, stunting—height-for-age *z* scores (HAZ) < −2—affects one in four preschool children.^1^ Stunting in early life has been linked to poor health and cognition, as well as reduced educational attainment and lower adult earnings.^2^–^5^ The reduction of stunting is therefore seen as an increasingly important long-term investment.^4^,^6^ Strategies for stunting reduction principally focus on improving infant and young child feeding (IYCF; including dietary diversification), and on preventing infections that reduce appetite, inhibit the absorption of nutrients and divert resources away from growth and development.

In the developing world, livestock ownership is regarded as an important means of diversifying diets through its role in income generation, as well as through directly improving household access to nutrient-rich animal-sourced foods.^7^–^10^ However, it has also been posited that exposure of children and their caregivers to animal feces is a potential risk factor for infections. A recent systematic review of 27 prior studies linking animal exposure to diarrhea symptoms or pathogens found that 20 studies showed significant positive linkages.^11^ In most of these studies, only a small proportion set out to specifically test diarrheal associations with animal exposure, only nine reported on their methods of recording animal exposure, and the body of evidence overall was determined to be of low quality. In addition to limited attention from researchers, strategic thinking and programming on diarrheal control has also primarily focused on reducing exposure to human rather than animal excreta,^12^ since humans are the main repository for several pathogens most strongly associated with diarrheal illnesses in children.^13^

Compared with diarrheal illnesses, even less attention has been focused on whether exposure to animal feces might affect child anthropometric outcomes such as height for age (stunting) or weight for height (wasting). Although there is a well-recognized bidirectional relationship between undernutrition and diarrheal illnesses, a growing body of research suggests subclinical environmental enteric disorder (EED)—rather than clinical diarrhea—is the primary causal pathway from poor sanitation and hygiene to stunting.^14^–^17^ EED is characterized by chronic damage to the small intestine, which inhibits the absorption of nutrients, but also triggers a low-level immune system stimulation (inflammation) that diverts resources away from physical and cognitive development, and leaves children more exposed to infections. Some of this research also hypothesizes that while the more pathogenic bacteria in human excreta may be the more important cause of diarrheal illnesses in young children, animal excreta may be an important reservoir of nonpathogenic bacteria that are still capable of causing the chronic subclinical damage to the gut, which is characterized as EED.^15^

In this regard, a particular concern is the possibility that young children in the developing world frequently ingest animal feces or fecally contaminated soils. Studies in Peru,^18^ Zimbabwe,^19^ and Bangladesh^20^ observed young children over prolonged periods and found that significant proportions ingested soils or poultry feces directly, whereas 28% of mothers in the Bangladesh study reported at least one incident of geophagy in the past week. All three studies showed that chicken feces and fecally contaminated soils had extremely high concentrations of bacteria, including pathogenic bacteria such as *Escherichia coli*. In a sample of 216 children under 5 years of age, the Bangladesh study found that the odds of being stunted (HAZ < −2) were double for children with caregiver-reported geophagy. Another study by the Bangladesh research team found that biomarkers of EED for these children were significantly associated with having an animal corralled in the child's sleeping room.^21^

Collectively, these studies suggest that the combination of free roaming livestock and poor hygiene and care practices may be an important underrecognized risk factor for EED and linear growth retardation. Nevertheless, the evidence to date is limited to the aforementioned study in Bangladesh,^20^ and experimental evidence on these linkages is unlikely to emerge for several more years.^22^,^23^

In this study, we therefore use data from large-scale nutrition surveys conducted in Ethiopia, Bangladesh, and Vietnam to address three research questions. First, within these quite different socioeconomic contexts, how prevalent are observable animal feces in household compounds? Second, what factors are most strongly associated with observable animal feces? Third, is the presence of animal feces significantly associated with child height for age (our primary outcome of interest), child weight for height, and child morbidity symptoms?

## Methods

### Data source and study population.

The data for this study are drawn from baseline and endline surveys conducted in rural areas of Bangladesh, Ethiopia, and Vietnam in 2010 and 2014 as part of a large project (Alive and Thrive) aimed at reducing undernutrition and death caused by suboptimal IYCF practices.^24^ Although these surveys have experimental designs for assessing the impacts of IYCF interventions, the present study cannot make use of these designs because the interventions did not directly address the nutritional risks associated with animal feces. The data can therefore be considered observational in nature. Nevertheless, these surveys are advantageous for the objectives of this study insofar as they include high-quality data on child anthropometry, morbidity symptoms and their determinants, including hygiene spot-checks for the presence of animal feces in the homestead compound as well as more standard indicators of household hygiene. The surveys contain large samples in each country, and the availability of multicountry data allows us to explore the question of external validity, and to specifically assess the potential scale of any health risks linked to exposure to animal feces. Since we are primarily interested in infants who are more likely to be left on the ground by themselves, and more likely to engage in exploratory mouthing behaviors or geophagy, we only use the subsample of children 6–23.9 months of age. As a result, data on anthropometry and hygiene indicators were available for 2,214, 1,750, and 2,104 mother and child dyads in Bangladesh, Vietnam, and Ethiopia, respectively. Ethical approval was obtained from the institutional review boards of the designated countries and from the International Food Policy Research Institute.

### Outcomes.

The primary outcome in this study was child anthropometry. Weight and supine length measurements were taken by trained fieldworkers using recommended protocols and standardized weighing scales and length boards accurate to 0.05 kg and 0.1 cm.^25^ Children's weight and length measurements were then used to derive length or HAZ and weight-for-length *z* scores (WHZ) by comparing each child's anthropometric measurements to the World Health Organization (WHO) child growth standards for his/her age and gender.^26^

A secondary set of outcomes pertained to child illness, which was measured through maternal recall of symptoms of diarrhea (three or more loose stools passed in a 24-hour period), as well as fever and cough/cold in the 2 weeks before the survey.

### Independent variables.

Our main independent indicators are observed animal feces; cleanliness of the child, mother, and the house; toilet use; improved water; and livestock ownership. Observations of animal feces and other cleanliness indicators were assessed via spot-check observations by survey enumerators, a method that has been used widely for the assessment of markers of hygiene practices.^27^ This method consists of observing a list of predetermined markers of hygiene practices on a single visit to a household. The interviewers were trained to record their observations related to the presence of animal feces around the house and key aspects of the cleanliness of the mother and her child (hair, hands, faces, and clothing) on a 3-point scale of clean, dusty, or dirty. We created dichotomous variables for mother or child cleanliness based on all four aspects being recorded as clean. Observations of household cleanliness were made based on a yes/no observation of key markers of inside and outside household hygiene, namely no human feces or garbage observed around the household compound, no dirty clothes inside the house, and the presence of a cover on the main drinking water container.

The other independent variables of interest were recorded directly from interviews with mothers. The survey also asks mothers about ownership of different types of livestock. For examining links with the presence of animal feces, we measure livestock types separately, but for our regression models, we aggregate these into a single index of tropical livestock units. We also defined improved toilet and water categories according to WHO definitions.^28^ Improved toilets include flush toilets, ventilated improved pit latrines, and pit latrines with slabs. Improved drinking water includes piped water, public tap or standpipe, tube well or borehole, and protected dug well or spring (public or private).

### Covariates.

Our models also control for a wide array of child, maternal, and household factors that are known to be associated with child anthropometry/undernutrition or child illness. Child factors included child's gender, age, and age square (for anthropometry outcomes). Maternal factors included mother's age, height, occupation, and education. Different cutoffs were used for maternal education for the three countries because education levels varied significantly. For example, in Vietnam, “primary education” was used as the reference group because “no schooling” is very rare, whereas in Bangladesh and Ethiopia, “no schooling” was used as the reference group. Likewise, very few mothers had advanced education levels in Ethiopia, so we created a category for any education beyond primary. In Bangladesh, few mothers had tertiary education so these mothers were pooled with those having secondary education.

At the household level, the models included household socioeconomic status (SES), self-reported food security, and number of children under 5 years of age. An SES index was constructed using principal components analysis with variables on ownership of house and land, housing quality, access to services, and household assets.^29^,^30^ Component scores derived from the first component (which explain 51%, 38%, and 31% of variance in Bangladesh, Ethiopia, and Vietnam, respectively) were then used to characterize the SES of each household. We also used a subjective assessment of whether the household could be classified as food secure based on Food and Nutrition Technical Assistance/U.S. Agency for International Development's Household Food Insecurity Access Scale.^31^ Finally, enumerators were also asked to record whether the household had experienced rainfall in the last 24 hours, since rainfall may mask the presence of feces in the compound, or influence other hygiene spot-check measures.

### Statistical analyses.

To explore external validity issues, these data were analyzed for each country separately and in regression models that pooled data from all three countries. Statistical analysis was implemented with STATA version 14 (Stata Corp., College Station, TX). Descriptive analyses were conducted to examine the different background characteristics of the study samples, particularly differences in nutrition and hygiene profiles across countries. We then use multivariate logit models to examine the associations between observed animal feces and various child, maternal, and household characteristics, including ownership of different types of livestock. In our main regression models, the primary outcome of interest are continuous anthropometric indicators (HAZ, WHZ), which we analyze with ordinary least squares models. Since the child morbidity variables are inherently dichotomous, we used logit regressions to analyze these health risks, but note that coefficient estimates from these models may suffer from greater imprecision.

For all our regression models, we used both unadjusted (bivariate) and adjusted (multivariate) regression models that control for a range of potentially confounding factors. The unadjusted models were estimated for six dependent variables of primary interest (animal feces, ownership of animals, maternal and child cleanliness, toilet use, and improved drinking water) to facilitate comparisons across these different water, sanitation, and hygiene (WASH)/livestock indicators. The adjusted models simultaneously include all six of these indicators, as well as an extensive array of covariates described above: child age, age square, and gender; maternal age, height, occupation, and education; household-level food security, SES, and number of children < 5 years of age; village rainfall and inclusion in the IYCF treatment group (in case of hygiene spillovers, which were emphasized more explicitly in the Bangladesh treatment). We also estimate a model based on pooling the data across all three surveys. In this model, all control variables are interacted with country dummies, but the coefficients on the main WASH and livestock indicators of interest are pooled across countries, and therefore reflect the weighted average coefficients.

In all the regressions, standard errors were clustered at the level of the enumeration area and significance levels were determined with one-sided tests on the primary coefficients of interest to reflect the a priori expectation that better hygiene outcomes should predict better nutrition and health outcomes, and that livestock ownership should be positively associated with nutrition and health.

## Results

### Descriptive statistics.

**Figure 1.**
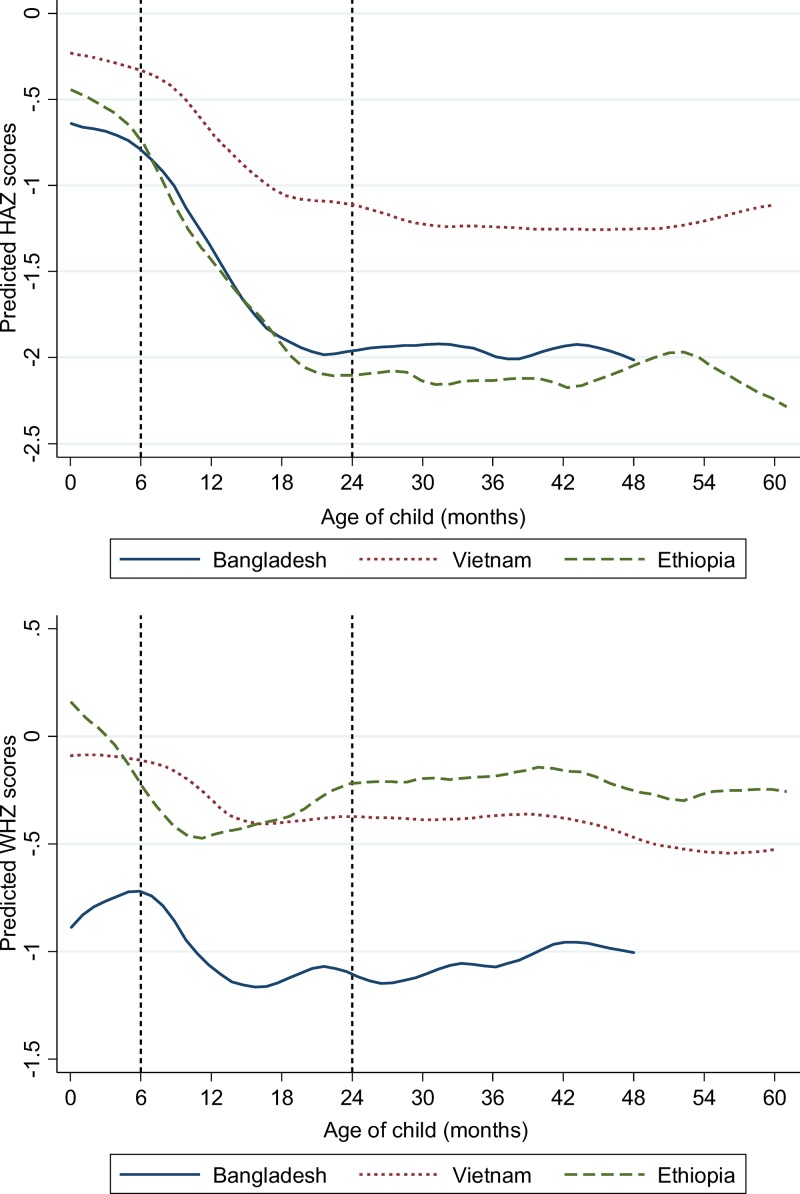
Height-for-age *z* scores (HAZ) and weight-for-length *z* scores (WHZ) by age in Bangladesh, Ethiopia, and Vietnam. All figures are estimated using the local polynomial smoother (lpoly command in STATA v14). HAZ: Bangladesh, *N* = 2,189; Vietnam, *N* = 2,098; Ethiopia, *N* = 1,689. WHZ: Bangladesh, *N* = 2,191; Vietnam, *N* = 2,096; Ethiopia, *N* = 1,710.

Table 1 reports characteristics of the study sample by country. Mean HAZ scores are very low in Ethiopia (−1.62) and Bangladesh (−1.58), but much higher in Vietnam (−0.83). Rates of stunting (HAZ < −2) follow similar patterns. In all three countries, linear growth faltering appears to begin in utero, but accelerates mostly rapidly in the 6- to 20-month period (Figure 1

), which includes the age range in which infants become more independent, are more likely to be left to sit or crawl alone, more likely to put objects in their mouth, and more likely to be fed potentially contaminated foods and liquids.

Patterns for wasting are somewhat different. As with HAZ scores, WHZ scores are relatively high in Vietnam (−0.33), but also quite moderate in Ethiopia (−0.40), whereas they are very low in Bangladesh (−1.05) where children tend to be born wasted (Figure 1). The data on morbidity symptoms show that diarrhea in the past 24 hours was most common in Ethiopia, but fever and cough/cold were more common in Bangladesh (over 40%), though still common in Ethiopia and Vietnam (about 20% in both countries). However, we note that the prevalence of these morbidity symptoms is likely to be quite seasonal.

There are other significant differences across samples. Ethiopia is substantially less developed than the other two countries, with lower education levels, lower rates of self-reported food security, and higher fertility rates (as reflected by more children under 5 years of age). Vietnam is at the other extreme, with many women having much higher levels of education and working outside of the homestead. Bangladesh lies in the middle of the other two countries, with relatively high rates of maternal education and low fertility rates, but relatively few mothers working outside the home.

Table 2 reports results specifically for the hygiene module, including our constructed variables such as “Mother fully clean,” “Child fully clean,” and “House fully clean,” as well as livestock ownership. Livestock ownership is very common in all three countries, particularly Ethiopia. Interestingly, poultry ownership is similar in all three countries, with approximately two-thirds of households rearing poultry. In retrospect, at least, it is therefore unsurprising that enumerators observed animal feces in the compound for around 40% of all households in each of the three countries. Hence, potential child exposure to animal feces is widespread, though actual exposure would depend on unobserved child care practices, which may indeed differ substantially across countries.

In contrast to animal feces, human feces were rarely observed in household compounds, except in Ethiopia where it was observed in 15.8% of households, and where almost one-third of households reported not owning a toilet. Even larger differences between countries are observed for other hygiene dimensions. Only about one-third of mothers and children in Ethiopia were classified as fully clean, whereas two-thirds of mothers in Bangladesh and Vietnam were classified as clean. Houses and surrounding compounds were rarely classified as fully clean across all eight dimensions, and there are diverse cross-country patterns across these indicators, which may be more affected by the subjective impressions of the enumerators. However, more objective measures suggest that hygiene levels in Vietnam are significantly better than Bangladesh or Ethiopia. In addition to the aforementioned differences in toilet use (almost universal in Vietnam), almost all Vietnamese mothers used soap for cleaning hands (95.7%), whereas this was much rarer in Bangladesh (44.0%) and Ethiopia (60.6%).

### Associations between the presence of animal feces and other household characteristics.

Table 3 uses logit regressions to identify some of the factors associated with exposure to animal feces. As expected, observations of animal feces are strongly associated with livestock ownership. Poultry ownership is robustly correlated with observed animal feces across all three countries, though the odds ratios vary from a low of 1.31 in Ethiopia to a very high 2.80 in Vietnam. Cow/buffalo ownership also has significant associations with observable animal feces, especially in Ethiopia where cattle ownership is very common (81.2%). Goat/sheep ownership only shares a marginal significant association in Bangladesh.

Observable animal feces probably also reflects general household hygiene standards, since more hygienic families may keep animals out of the household compound, or may remove livestock feces from the homestead area. Indeed, there are highly significant and robust associations between observed animal feces and maternal and child cleanliness (with odds ratios that vary between 0.52 and 0.71), although there are no significant associations with toilet or water facilities, except in the case of hygienic toilets in Vietnam. One might also expect most forms of hygiene to be related to parental education or SES. However, results show that the associations vary in sign and significance across countries. SES and maternal education are unrelated to observed animal feces in Bangladesh. In Ethiopia, higher SES households are more likely to have animal feces observed in their compound (presumably because SES is strongly associated with livestock ownership), but more educated Ethiopian mothers are less likely to have animal feces in their compounds. In Vietnam, the poorest households are more likely to have animal feces in their compounds, but maternal education is not significantly associated with the presence of animal feces.

In summary, the results in Table 3 point to livestock ownership and poor personal cleanliness as being strongly associated with the presence of animal feces. This also highlights the importance of multivariate tests that control for these other dimensions of hygiene, since these are potentially confounding factors that may bias bivariate tests.

### Associations between the presence of animal feces and child anthropometry.

Table 4 reports results from unadjusted and adjusted least squares regression models for HAZ and WHZ scores.^15^ In Bangladesh and Ethiopia, the observed presence of animal feces in the compound is significantly and negatively associated with child HAZ scores in both the unadjusted and adjusted models. The coefficient in the Ethiopian sample (−0.22) is somewhat larger than that of the Bangladesh sample (−0.13), though these coefficients are not statistically different to each other. In Vietnam, we did not find any significant association between anthropometric outcomes and animal feces in the unadjusted or adjusted models, nor were there significant associations with any of the other hygiene indicators in the adjusted HAZ model for Vietnam, though child cleanliness has a significant coefficient in the WHZ model. A pooled model with interactions between animal feces and country dummy variables also shows that the coefficients on animal feces in Ethiopia and Bangladesh are significantly different to the coefficient for Vietnam.

In addition to the associations with animal feces, the coefficient on tropical livestock units is of interest, since this indicator may capture the presumably positive effects that livestock may have on income/wealth and animal-sourced food consumption. However, we only observe a significantly positive coefficient in Ethiopia, where livestock ownership is very high because of greater rates of cattle ownership, which has been significantly linked to child HAZ scores.^10^

Among the other hygiene indicators, the only significant coefficients among the adjusted HAZ models pertain to hygienic toilets and water sources in Bangladesh. The coefficients on these two variables are again similar in magnitude to the coefficient on animal feces in the Bangladesh HAZ model, and we cannot reject the null hypothesis that these coefficients are equal in absolute magnitude.

For WHZ scores, we find significant associations with animal feces only in the unadjusted models for all three countries. Once the full set of controls is included, the coefficient on animal feces is insignificant in all countries. However, most hygiene indicators also yield insignificant coefficients in these adjusted WHZ models, with only two exceptions (improved toilets in Bangladesh and child cleanliness in Vietnam). In Ethiopia, tropical livestock units are again significant in the WHZ model.

### Associations between the presence of animal feces and child morbidity symptoms.

Associations between hygiene indicators and recent symptoms of child illness are presented in Table 5. In the diarrhea models for Bangladesh and Vietnam, the unadjusted odds ratio on observed animal feces are large and significant at the 5% level or more, but the corresponding odds ratios in the adjusted model is only significant at the 10% level for Bangladesh and is not significant in Vietnam. The odds ratio in Bangladesh suggests that the presence of animal feces increases the risk of diarrhea by 25%. Animal feces does not yield any significant coefficients in the regression models for fevers or cough/cold. There are also few other WASH or livestock coefficients that are statistically significant in Table 5, though maternal cleanliness yields significant coefficients in the fever and cough/cold models for Ethiopia, and tropical livestock units is associated with lower odds of fever incidence in Ethiopia. In the pooled models, the coefficient on animal feces is not significant in explaining any of the three morbidity indicators. Only maternal cleanliness continues to have some explanatory power in the fever and cough/cold regressions, whereas tropical livestock units are associated with lower odds of fever symptoms.

## Discussion

Previous studies have often found positive associations between livestock ownership and child growth,^10^,^32^–^34^ though several have found insignificant associations.^35^,^36^ It is possible that livestock ownership has positive effects on child growth through improvements in household SES, animal-sourced food consumption, and other mechanisms (transportation, social status, women's empowerment, collateral for credit), but also negative impacts through increased risk of infections and/or EED.

Consistent with an infection/EED pathway, our data from Bangladesh and Ethiopia reveal that the presence of animal feces in the compound is negatively associated with child HAZ. In Ethiopia, we also found that livestock ownership was positively associated with child HAZ outcomes, as has previous research.^10^ Interestingly, we did not find analogous associations in the adjusted HAZ model for the Vietnam sample. We hypothesize two possible explanations. First, lack of an association might be due to Vietnam having much better nutrition in general (Table 1), which could indicate that EED or repeated diarrheal infections are less prevalent in Vietnam. Notably, all the other WASH indicators also yielded insignificant coefficients in the HAZ regressions for Vietnam. Second, it is likely that Vietnamese children are exposed to much better care practices than children in Ethiopia or Bangladesh. We observed that handwashing with soap is almost universal in Vietnam (but much less common in the other two countries), and it may also be that Vietnamese children are rarely left unattended on floors. These better practices would therefore provide a barrier between the animal feces and ingestion of fecal matter by small children.

Our findings for Ethiopia and Bangladesh are consistent with two recent studies analyzing a smaller (*N* = 214) sample of children in Mirzapur, subdistrict of Bangladesh, which found significant links between geophagy, animal exposure, EED, and stunting.^20^,^21^ However, these earlier results from Bangladesh were based solely on poultry-owning households and the regression models included very few controls (only child age, gender, maternal education, and household size); so, it is encouraging that the results in this article reach similar conclusions with a different measure (observed animal feces instead of animal proximity to a child), a much larger sample, and a more comprehensive set of control variables.

We found weaker associations between observed animal feces and the prevalence of morbidity symptoms, though we did observe a marginally significant odds ratio in the diarrhea model for Bangladesh. This positive association between animal feces and diarrhea is consistent with a recent meta-analysis uncovering linkages between animal ownership and diarrhea.^11^ However, that analysis also concluded that associations between diarrhea and exposure to animals were stronger among studies that elucidated and confirmed a microbial cause of diarrhea through laboratory methods, whereas studies using morbidity symptoms were less likely to uncover a significant association.^11^ Hence, our models arguably constitute weak tests because of well-known flaws with morbidity measures based on parental reports of recent symptoms only.

Our findings contribute to a growing body of evidence suggesting that child anthropometry and health outcomes in developing countries may be adversely affected by exposure to animals and their feces. This risk stems from several factors: from the widespread ownership of livestock and pets in developing countries, from the lack of housing and enclosure structures for livestock that separate animals from household members (e.g., scavenging poultry systems), from poor hygiene knowledge and practices, from the common practice of leaving children to sit or play on homestead floors with little monitoring (especially in warmer climates), and of course from the very high concentration of potentially harmful bacteria in animal feces.

On the first of these risk factors, we know that livestock ownership is extremely common in our sample. Over 60% of households own poultry in all three countries, and ownership of other livestock is also very common, especially in Ethiopia. However, such high rates of livestock ownership are hardly unique to these three countries. Among Demographic Health Surveys for rural areas of 47 developing countries with the requisite data, average ownership of any kind of livestock was 68%, with poultry by far the most common type (52%). In 10 of the 47 countries, livestock ownership exceeded 80% among rural households.

On the second of the aforementioned risk factors, there is no systematic measurement of whether livestock are kept in enclosures, but poultry, in particular, are typically allowed to freely roam around and even within the house in search of household food waste, typically stay within 50 m of the main house at all times, and are often kept within the house overnight.^37^ In the aforementioned Zimbabwe study, researchers reported observing poultry within the main household dwelling itself, even around food preparation areas.^19^ In Bangladesh, 14% of poultry-owning household corralled poultry in the same room where children slept. In a recent survey in rural Ethiopia, researchers found that 48% of poultry-owning households kept their animals in the main household dwelling overnight.^38^ Combined with other poor hygiene practices—such as limited handwashing—this elevated exposure to the feces of poultry and other livestock may well produce a significant risk of infection, EED, and linear growth retardation.

Although the findings in this study are strongly suggestive of an important child health risk linked to livestock ownership, this study has several limitations. Hygiene spot-checks of animal feces in the exterior of the compound serve only as rough proxies of exposure to animal feces. Ideally, we need to record whether feces are regularly present in the compound, what types of animal feces are prevalent in the compound, whether feces were present inside the main household dwelling, whether children were left on homestead floors unattended, and whether children had ever been observed to handle, mouth, or ingest livestock feces. Moreover, geophagy may not be the only vector linking animal exposure to health outcomes. For example, a recent microbiological study of 24 rural villages conducted in Odisha, India, found that animal fecal matter was present in 83% of public water sources.^39^ Finally, although the Alive and Thrive surveys are advantageous in allowing us to control for a range of socioeconomic and hygiene factors that one would expect to be associated with both the presence of animal feces and nutrition outcomes (Table 3), it is still possible that our results are biased by unobserved confounding factors. In short, there is clearly ample scope for future research to improve the measurement of exposure to animal feces, to explore biological mechanisms linking this exposure to EED, morbidity symptoms, and child anthropometric outcomes, and to experiment with livestock and WASH interventions that can influence these linkages.

These caveats aside, our results add to a growing body of evidence suggesting that child growth outcomes are significantly influenced by exposure to domesticated animals and their feces. This literature also points to the need to reevaluate several core features of conventional WASH strategies. First, most WASH strategies have focused on influencing diarrhea and morbidity outcomes, even though impacts on nutrition may be large and manifested via subclinical EED. Second, most WASH strategies have focused behavioral change communications on a small number of key messages, with particular emphasis on reducing exposure to human feces.^12^ This approach rests on the assumption that exposure to human feces poses a greater health burden (to children especially) than exposure to animal feces. However, such an assumption seems increasingly contestable, particularly in the context of nutrition outcomes.^12^ Indeed, although it is certainly the case that open defecation remains a major health concern in much of the developing world, exposure to animal feces is probably more common, and potentially also hazardous for child nutrition and health outcomes. Behavioral change communications strategies should therefore consider reducing exposure to all excreta, human and animal, and aspire to genuine “total sanitation.”

## Figures and Tables

**Table 1:** Selected characteristics of the study sample in Bangladesh, Ethiopia, and Vietnam

	Bangladesh (*N* = 2,214)	Ethiopia (*N* = 1,750)	Vietnam (*N* = 2,104)
Health outcomes			
Child anthropometry			
HAZ	−1.6 ± 1.4	−1.6 ± 1.6	−0.8 ± 1.1
WHZ	−1.1 ± 1.2	−0.4 ± 1.2	−0.3 ± 1.1
Stunting (%)	38.0	41.3	13.9
Wasting (%)	19.5	8.0	4.4
Child morbidity			
Diarrhea (%)	8.8	20.1	9.8
Fever (%)	41.5	26.4	26.5
Cough/cold (%)	41.2	31.3	28.7
Maternal characteristics			
Age (years)	26.0 ± 5.8	28.3 ± 6.3	28.7 ± 5.4
Education			
No schooling (%)	21.0	58.6	
Primary school (%)	29.6	25.9	10.2
Secondary school or higher (%)	49.5	15.5	47.5
High school or higher (%)			42.4
Occupation of mother			
Farmer or housewife (%)	87.5	91.0	47.8
Other (%)	12.5	9.0	52.2
Height (m)	1.5 ± 0.1	1.6 ± 0.1	1.5 ± 0.1
Child characteristics			
Age (months)	14.8 ± 5.2	14.2 ± 5.2	14.7 ± 5.4
Child (female) (%)	49.1	48.8	47.0
Household characteristics			
Household food security (%)*	74.5	39.5	68.5
Household SES†			
Lowest tercile (%)	33.3	34.1	33.4
Middle tercile (%)	33.3	32.8	33.3
Highest tercile (%)	33.3	33.1	33.3
No. of children < 5 years of age (%)			
1 child (%)	77.1	52.9	74.7
≥ 2 children (%)	22.9	47.1	25.2

HAZ = height-for-age *z* scores; SES = socioeconomic status; WHZ = weight-for-length *z* scores.

* Household food security was measured using Food and Nutrition Technical Assistance/U.S. Agency for International Development's Household Food Insecurity Access Scale.

† An SES index was constructed using principal components analysis with variables on ownership of house and land, housing quality, access to services, and household assets. The SES index was then divided into terciles.

**Table 2:** Hygiene spot-check observations in Bangladesh, Ethiopia, and Vietnam

	Bangladesh (*N* = 2,214)	Ethiopia (*N* = 1,750)	Vietnam (*N* = 2,104)
Owns any livestock (%)	73.7	86.2	74.4
No. of chickens (%)			
0	37.3	39.9	31.3
1–10	48.4	58.8	26.9
≥ 11	14.3	1.3	41.8
No. of cattle/buffalo (%)			
0	64.5	23.5	71.7
1–2	24.4	38.1	28.3
≥ 3	11.2	38.4	0.0
No. of goat/sheep (%)			
0	77.0	62.0	99.4
≥ 1	30.0	38.0	0.6
Tropical livestock unit (mean ± SD)	0.7 ± 1.2	2.0 ± 2.2	1.0 ± 2.1
Animal feces in compound (%)	40.6	37.9	41.7
Improved toilet* (%)	28.8	1.0	49.2
Any toilet* (%)	96.0	82.7	95.1
Use of soap for hand cleaning (%)	44.0	60.6	95.7
Improved drinking water† (%)	68.8	66.5	86.9
Cleanliness of the mother (4 items)			
Hands (%)	84.4	63.5	72.6
Hair (%)	85.2	57.7	95.0
Clothes (%)	74.6	39.0	84.7
Face (%)	88.7	80.3	95.7
Mother fully clean‡ (%)	72.8	34.4	69.0
Cleanliness of the child (4 items)			
Hands (%)	70.9	60.4	75.4
Hair (%)	78.0	68.0	93.4
Clothes (%)	65.1	36.4	81.3
Face (%)	75.3	68.7	90.4
Child fully clean‡ (%)	62.6	32.7	70.2
Cleanliness of the house (8 items)			
Clean general appearance of compound (%)	58.8	52.8	43.7
Area around house does not need cleaning (%)	53.7	44.7	76.8
Human feces not around (%)	95.2	84.2	99.0
No garbage in the compound (%)	52.3	79.3	36.2
Clean interior of house (%)	72.4	53.1	65.0
Clean floor (%)	65.6	47.3	56.0
Covered drinking water (%)	49.3	71.8	66.4
No piles of dirty clothes (%)	62.9	39.6	54.8
House fully clean§	24.9	15.8	20.1

SD = standard deviation.

* Improved toilet follows the World Health Organization (WHO) definition,^28^ and is defined as flush toilets, ventilated improved pit latrines, or pit latrines with slabs.

† Improved toilet follows the WHO definition,^28^ and includes piped water, public tap or standpipe, tubewell or borehole, protected dug well or spring (public or private).

‡ Child and mother “fully clean” refers to all four items being classified as clean (not dirty or dusty) in each case.

§ House fully clean refers to all eight household hygiene items being classified as clean.

**Table 3:** A logit model explaining the presence of animal feces in the compound

	Bangladesh	Ethiopia	Vietnam
No. of chickens (0 as reference)			
1–10	1.55** (0.20)	1.31* (0.16)	2.33*** (0.42)
≥ 11	2.17*** (0.41)		2.80*** (0.46)
No. of cattle/buffalo (0 as reference)			
1–2	1.34* (0.19)	3.52*** (0.67)	1.59*** (0.24)
≥ 3	1.45* (0.24)	2.76*** (0.61)	
No. of goat/sheep (0 reference)			
≥ 1	1.31+ (0.18)	1.07 (0.14)	0.63 (0.38)
Mother fully clean	0.60*** (0.08)	0.52*** (0.08)	0.50*** (0.07)
Child fully clean	0.60*** (0.06)	0.68** (0.10)	0.71* (0.11)
Improved toilet	0.90 (0.14)	0.68 (0.34)	0.58*** (0.08)
Improved drinking water	0.99 (0.12)	0.88 (0.15)	0.77 (0.21)
SES (lowest reference)			
Low	1.05 (0.17)	0.75 (0.16)	0.53*** (0.09)
Middle	1.11 (0.12)	1.10 (0.26)	0.78 (0.16)
High	0.82 (0.18)	1.80* (0.44)	0.63** (0.14)
Highest	0.78 (0.16)	3.31*** (0.85)	0.56** (0.14)
Mother's education			
Primary school	0.95 (0.13)	0.88 (0.10)	
Middle school	1.00 (0.14)	0.66* (0.12)	0.90 (0.18)
High school or higher			0.74 (0.16)
Dummy for 2014 round	0.58*** (0.09)	1.11 (0.19)	0.05*** (0.01)
*N*	2,214	1,692	2,098

SES = socioeconomic status. Significance levels are reported for two-sided tests: + *P* < 0.10, * *P* < 0.05, ** *P* < 0.01, *** *P* < 0.001.

**Table 4:** Model for HAZ and WHZ among children 6–23.9 months in unadjusted and adjusted models

	Bangladesh		Ethiopia		Vietnam		Pooled
	Unadjusted†	Adjusted‡	Unadjusted†	Adjusted‡	Unadjusted†	Adjusted‡	Adjusted‡
HAZ§							
Animal feces	−0.26*** (0.06)	−0.13* (0.07)	−0.19* (0.08)	−0.22* (0.08)	−0.02 (0.05)	0.03 (0.06)	−0.11** (0.04)
Tropical livestock units	0.02 (0.03)	−0.04 (0.03)	0.04* (0.02)	0.07** (0.02)	−0.03* (0.01)	−0.02 (0.01)	0.01 (0.01)
Mother fully clean	0.37*** (0.07)	0.03 (0.07)	0.11 (0.08)	−0.06 (0.09)	0.16** (0.05)	0.00 (0.07)	−0.00 (0.04)
Child fully clean	0.43*** (0.06)	0.01 (0.05)	0.33*** (0.08)	0.11 (0.09)	0.27*** (0.05)	0.08 (0.07)	0.06 (0.04)
Improved toilet	0.40*** (0.07)	0.13** (0.04)	−0.35 (0.39)	−0.41 (0.26)	0.25*** (0.05)	0.07 (0.05)	0.10* (0.05)
Improved drinking water	0.42*** (0.06)	0.19* (0.07)	−0.09 (0.08)	−0.10 (0.10)	0.28*** (0.07)	0.13 (0.08)	0.07+ (0.04)
*N*	2,189	2,188	1,680	1,658	2,098	2,095	5,861
WHZ¶							
Animal feces	−0.12* (0.05)	−0.07 (0.05)	−0.14* (0.06)	−0.03 (0.06)	−0.24*** (0.05)	0.00 (0.06)	−0.03 (0.03)
Tropical livestock units	0.03 (0.02)	0.01 (0.02)	0.03* (0.01)	0.06** (0.02)	0.002 (0.01)	0.00 (0.01)	0.02* (0.01)
Mother fully clean	0.21*** (0.06)	0.05 (0.07)	0.16* (0.06)	0.09 (0.07)	0.28*** (0.05)	−0.01 (0.06)	0.05 (0.04)
Child fully clean	0.18*** (0.05)	−0.01 (0.06)	0.16** (0.06)	0.12 (0.08)	0.36*** (0.05)	0.21** (0.06)	0.11** (0.04)
Improved toilet	0.26*** (0.06)	0.15* (0.07)	−0.35 (0.30)	−0.25 (0.23)	0.14** (0.05)	−0.08 (0.06)	0.04 (0.04)
Improved drinking water	0.16** (0.06)	0.05 (0.07)	−0.06 (0.06)	−0.06 (0.07)	0.23*** (0.07)	0.02 (0.07)	0.01 (0.04)
*N*	2,189	2,188	1,680	1,658	2,098	2,095	5,861

HAZ = height-for-age *z* scores; WHZ = weight-for-length *z* scores. + *P* < 0.10, * *P* < 0.05, ** *P* < 0.01, *** *P* < 0.001 reported for one-sided tests associated with the alternative hypothesis that better hygiene leads to better nutrition. All standard errors are clustered at the enumeration area level.

† Unadjusted model: bivariate analysis.

‡ Adjusted model includes the following variables: animal feces; tropical livestock units; cleanliness of mother and children; improved toilet and water sources; child age and child age square; mother's height, education, and occupation; number of children < 5 years of age; household socioeconomic status and food security; rain yesterday.

§ *R*^2^ for adjusted HAZ model: 19.4% for Bangladesh, 13.0% for Ethiopia, 17.1% for Vietnam, and 21.4% for pooled model.

¶ *R*^2^ for adjusted WHZ model: 4.8% for Bangladesh, 5.7% for Ethiopia, 7.0% for Vietnam, and 12.5% for pooled model.

**Table 5:** Logit models for child illness among children 6–23.9 months in Bangladesh, Ethiopia, and Vietnam

	Bangladesh		Ethiopia		Vietnam		Pooled
	Unadjusted†	Adjusted‡	Unadjusted†	Adjusted‡	Unadjusted†	Adjusted‡	Adjusted‡
Diarrhea							
Animal feces	1.45* (0.22)	1.25+ (0.17)	1.13 (0.14)	1.07 (0.16)	1.62** (0.24)	0.95 (0.19)	1.08 (0.10)
Tropical livestock units	0.93 (0.07)	0.93 (0.06)	0.96 (0.03)	0.97 (0.03)	0.91 (0.05)	0.95 (0.05)	0.96 (0.03)
Mother clean	0.68* (0.11)	0.85 (0.18)	0.61*** (0.08)	0.65* (0.12)	0.76+ (0.12)	1.23 (0.23)	0.85 (0.09)
Child clean	0.73* (0.11)	0.98 (0.16)	0.74* (0.10)	0.95 (0.18)	0.79 (0.12)	0.78 (0.17)	0.91 (0.09)
Improved toilet	0.66* (0.12)	0.87 (0.20)	1.66 (0.89)	1.72 (1.08)	0.83 (0.12)	0.92 (0.16)	0.94 (0.13)
Improved drinking water	0.71* (0.11)	0.87 (0.15)	0.69** (0.08)	0.76* (0.10)	0.88 (0.18)	1.13 (0.26)	0.86 (0.08)
*N*	2,214	2,214	1,735	1,623	2,100	2,094	
Fever							
Animal feces	1.20* (0.11)	1.05 (0.10)	1.06 (0.12)	1.16 (0.18)	1.54*** (0.15)	0.92 (0.11)	1.03 (0.07)
Tropical livestock units	0.92* (0.04)	0.94+ (0.04)	0.91** (0.03)	0.96 (0.03)	0.94* (0.03)	0.95 (0.04)	0.95* (0.02)
Mother clean	0.78* (0.08)	0.95 (0.13)	0.60*** (0.07)	0.62** (0.11)	0.65*** (0.07)	0.92 (0.11)	0.85* (0.06)
Child clean	0.82* (0.07)	1.06 (0.10)	0.78* (0.09)	0.99 (0.16)	0.77* (0.08)	1.01 (0.11)	1.02 (0.07)
Improved toilet	0.75** (0.07)	0.97 (0.08)	1.53 (0.78)	1.81 (0.84)	0.93 (0.09)	1.06 (0.13)	1.04 (0.08)
Improved drinking water	0.81* (0.07)	0.99 (0.14)	0.73** (0.08)	0.87 (0.12)	0.87 (0.12)	1.16 (0.15)	0.99 (0.07)
*N*	2,214	2,214	1,735	1,623	2,100	2,094	
Cough/cold							
Animal feces	1.02 (0.09)	0.86 (0.08)	0.99 (0.11)	1.14 (0.15)	1.41*** (0.14)	1.19 (0.15)	1.00 (0.07)
Tropical livestock units	0.96 (0.04)	0.99 (0.04)	0.91** (0.03)	0.97 (0.03)	0.96 (0.03)	0.96 (0.03)	0.97 (0.02)
Mother clean	0.70*** (0.07)	0.83 (0.11)	0.72** (0.08)	0.73* (0.11)	0.80* (0.08)	0.99 (0.12)	0.84* (0.06)
Child clean	0.78** (0.07)	0.97 (0.09)	0.84 (0.09)	0.98 (0.15)	0.85 (0.09)	1.03 (0.14)	1.00 (0.07)
Improved toilet	0.79* (0.08)	1.03 (0.11)	3.18* (1.57)	4.16+ (3.54)	0.93 (0.09)	0.93 (0.12)	1.03 (0.08)
Improved drinking water	0.79* (0.07)	0.92 (0.11)	0.74** (0.08)	0.82 (0.10)	0.79+ (0.11)	0.86 (0.15)	0.88+ (0.06)
*N*	2,214	2,214	1,735	1,622	2,100	2,094	

+ *P* < 0.10, * *P* < 0.05, ** *P* < 0.01, *** *P* < 0.001 reported for one-sided tests associated with the alternative hypothesis that better hygiene or more livestock units leads to a lower likelihood of morbidity symptoms. All standard errors are clustered at the enumeration area level.

† Unadjusted model: bivariate analysis.

‡ Adjusted model: model included animal feces; tropical livestock units; cleanliness of mother and children; improved toilet and water sources; child age and child age square; mother's height, education, and occupation; number of children < 5 years of age; household socioeconomic status and food security; rain yesterday.
